# Prognostic Impact of Late Gadolinium Enhancement on Major Adverse Cardiovascular Events and Time‐Varying Attenuation of Risk in Dilated Cardiomyopathy

**DOI:** 10.1002/clc.70506

**Published:** 2026-10-29

**Authors:** Liyuan Ma, Yuan Li, Huairong Zhang, Yan Zheng, Pei Wang, Yuanyuan Niu, Li Zhu

**Affiliations:** ^1^ Ningxia Medical University General Hospital Yinchuan China; ^2^ The First Clinical Medical College of Ningxia Medical University Yinchuan China; ^3^ Department of Radiology General Hospital of Ningxia Medical University Yinchuan China

**Keywords:** cardiovascular magnetic resonance, dilated cardiomyopathy, late gadolinium enhancement, prognosis

## Abstract

**Objectives:**

To investigate the prognostic value of late gadolinium enhancement (LGE) extent for major adverse cardiovascular events (MACE) in dilated cardiomyopathy (DCM) patients and characterize the dynamic pattern of its risk effect over time.

**Methods:**

We retrospectively enrolled patients who underwent Cardiac Magnetic Resonance (CMR) between 2020 and 2024, with MACE (cardiac death, heart failure re‐hospitalization, or sustained ventricular arrhythmia) as the endpoint. Patients were stratified by MACE occurrence. Schoenfeld residual testing showed borderline deviation of the proportional hazards (PH) assumption for LGE extent (LV mass percentage). Thus, a time‐dependent Cox model (Extended Cox PHs Model) with an LGE extent × follow‐up time interaction term was built to assess dynamic hazard ratio changes, and independent MACE risk factors were identified.

**Results:**

Among 173 DCM patients (age 47 ± 15 years, 105 males), 46 (26.6%) experienced MACE after a mean follow‐up of 27 ± 16 months. The time‐dependent Cox model identified LGE extent (baseline HR: 1.243, 95% CI: 1.162–1.329, *p* < 0.001), the LGE extent × time interaction (HR: 0.993, 95% CI: 0.989–0.997, *p* = 0.001), and LV end‐diastolic volume index (LVEDVi; HR = 1.008, 95% CI: 1.003–1.014, *p* = 0.001) as independent MACE predictors. Notably, the risk effect of LGE extent demonstrated time‐dependent attenuation.

**Conclusion:**

LV LGE extent and LVEDVi independently predict MACE in DCM patients. The early prognostic value of LGE extent suggests a critical time window for early fibrosis intervention.

## Graphical abstract

In 173 dilated cardiomyopathy patients, late gadolinium enhancement (LGE) independently predicted major adverse cardiovascular events, but its risk effect attenuated over ~34 months, while LVEDVi maintained stable long‐term prediction. Early CMR‐based risk stratification identifies a critical time window for intervention.

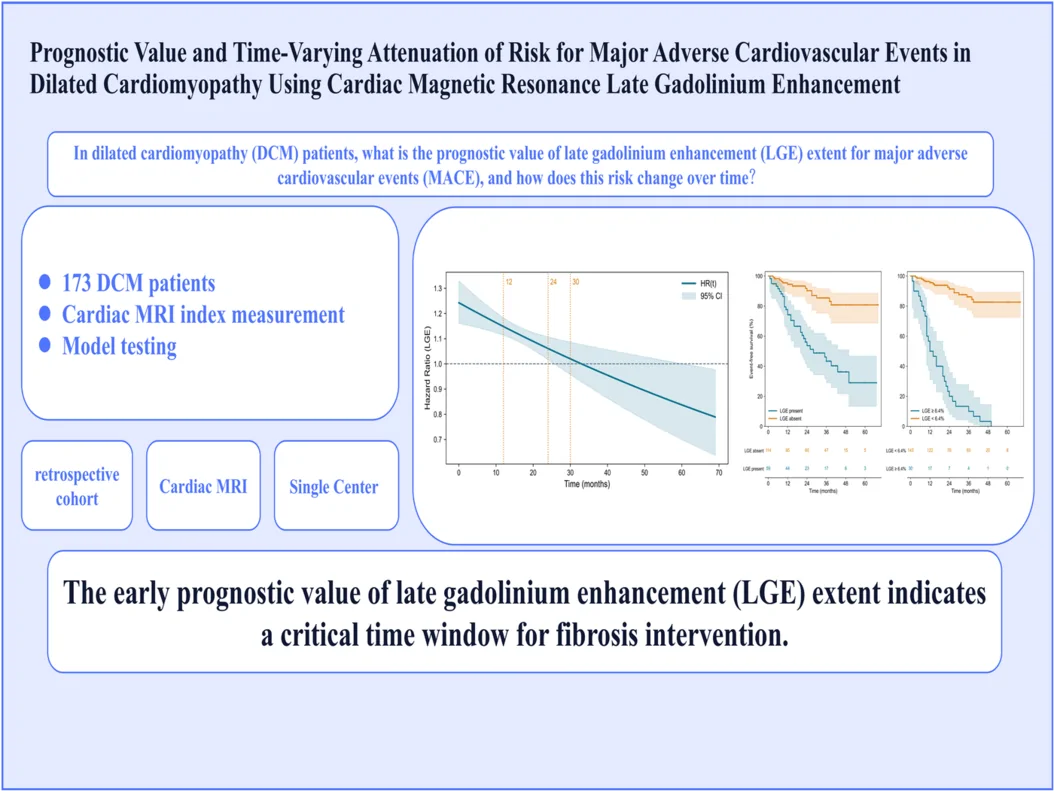

## 1 Introduction

Dilated cardiomyopathy (DCM) is characterized by ventricular dilation and progressive systolic dysfunction, with pathogenesis independent of coronary artery disease or abnormal hemodynamic loads (e.g., hypertension, valvular disease) [1, 2, 3]. DCM has an estimated prevalence of approximately 1 in 2500 adults. As a major cause of heart failure (HF) in young adults and the leading indication for heart transplantation worldwide, DCM carries a high 5‐year mortality rate of up to 20% [4, 5], primarily attributable to progressive HF or sudden cardiac death (SCD). Given its substantial incidence and mortality burden, DCM poses a significant challenge to global public health.

Current clinical guidelines utilize left ventricular ejection fraction (LVEF) and New York Heart Association (NYHA) functional class for risk assessment in patients [6]. While LVEF is considered a strong predictor of all‐cause mortality—with DCM mortality inversely correlating with LVEF—its discriminatory power diminishes in severe systolic dysfunction [7]. This suggests LVEF alone may fail to capture the complex pathophysiology in advanced DCM. Although guidelines recommend implantable cardioverter‐defibrillator (ICD) for primary SCD prevention in patients with HF and LVEF ≤ 35% (despite at least 3 months of guideline‐directed medical therapy) and NYHA functional class II–III [8], the DANISH trial series demonstrated no significant survival benefit from prophylactic ICD implantation in DCM patients with reduced LVEF [9, 10, 11]. This paradox reveals limitations in traditional LVEF‐based stratification for explaining DCM's heterogeneous pathology and prognostic variability.

Previous studies report that late gadolinium enhancement (LGE) strongly predicts SCD risk stratification in DCM patients with LVEF ≥ 35%, suggesting its inclusion in primary prevention therapy criteria (e.g., ICD implantation) [12, 13, 14]. However, current research predominantly focuses on binary LGE presence/absence [12], lacking dynamic analysis of how its risk effect evolves temporally [15]. This gap impedes precise identification of optimal intervention windows.

Therefore, we aimed to investigate both the predictive value of LGE extent for major adverse cardiovascular events (MACE) in DCM patients and its dynamic risk effect over time, thereby providing a novel indicator for clinical intervention timing.

## 2 Materials and Methods

### 2.1 Study Population

The Ethics Committee of the General Hospital of Ningxia Medical University approved the study protocol (Approval No.KYLL‐2023‐086). We retrospectively and consecutively enrolled patients with nonischemic DCM who underwent LGE cardiac magnetic resonance imaging (LGE‐CMR) at the General Hospital of Ningxia Medical University between January 2020 and June 2024. DCM diagnosis followed current European [2] and American guidelines [16], requiring fulfillment of the following CMR‐based criteria: left ventricular (LV) dilation (LV end‐diastolic dimension or volume) > 2 standard deviations (SD) above the population mean normalized for body surface area, sex, and/or age; and LVEF < 50%. Exclusion criteria included: Ischemic cardiomyopathy (coronary artery stenosis ≥ 50% in major branches on coronary CTA or angiography, history of myocardial infarction, coronary stenting/bypass grafting, or ischemic distribution pattern on CMR perfusion/LGE imaging); Secondary DCM due to hypertension, valvular disease, hypertrophic cardiomyopathy, congenital heart disease, or infiltrative cardiomyopathy; Absolute contraindications to CMR (claustrophobia, implanted devices, metallic heart valves, non‐CMR compatible vascular clips); Severe renal insufficiency, hepatic insufficiency, or bone metabolism abnormalities. Demographic data, medical history, and NYHA functional class were collected for all enrolled subjects.

MACE were primarily defined as cardiac death, first HF rehospitalization, or sustained ventricular arrhythmia: (1) Cardiac mortality: Death definitively attributed to cardiac causes, excluding non‐cardiac deaths (e.g., infection, malignancy, and trauma); (2) First HF rehospitalization: Hospitalization ≥ 24 h requiring intravenous diuretics, vasoactive drugs, or mechanical circulatory support due to worsening HF symptoms following DCM diagnosis [6]; (3) Sustained ventricular arrhythmia: Sustained ventricular tachycardia or non‐fatal ventricular fibrillation requiring medical treatment, electrical cardioversion, or appropriate ICD therapy. Hospitalizations for non‐HF reasons or elective procedures were excluded. All events were adjudicated by physicians blinded to CMR findings and confirmed through comprehensive medical record review, clinical follow‐up, and telephone interviews. Overall survival time was calculated from enrollment to endpoint occurrence or last follow‐up (Figure 1).

**Figure 1:**
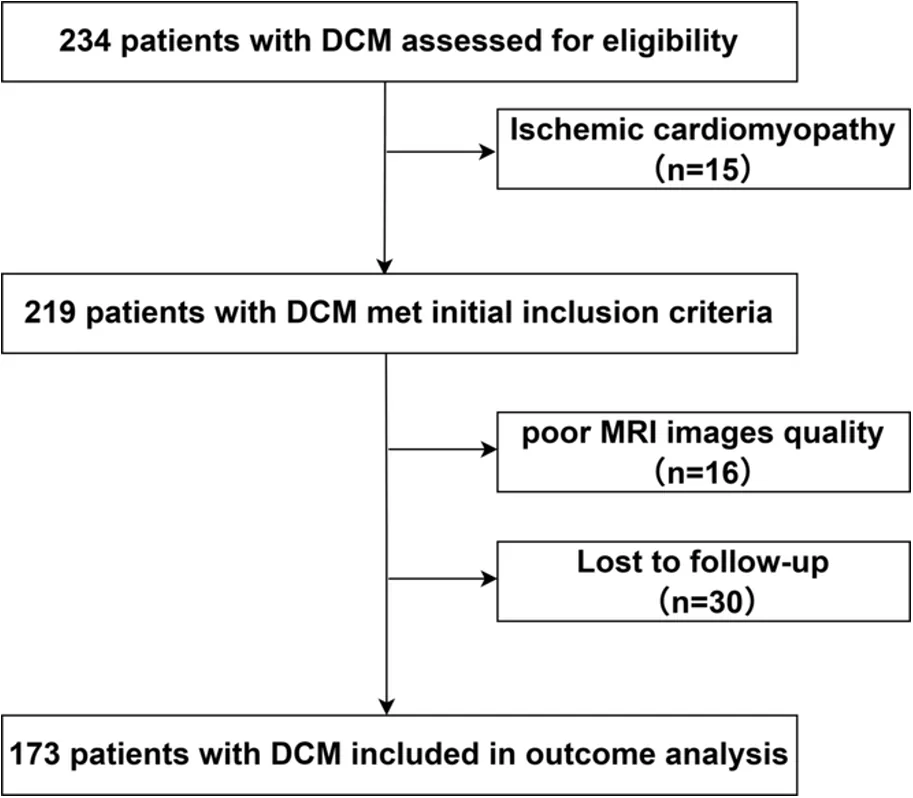
Flowchart shows patient inclusion. DCM, dilated cardiomyopathy.

### 2.2 Cardiac MRI Acquisition

All subjects underwent scanning using a 3.0 T superconducting MR scanner (Ingenia, Philips Healthcare, Netherlands). After removing metallic objects, subjects were positioned supine with an 8‐channel cardiac phased‐array coil placed anteriorly and posteriorly. Breath‐holding training was provided at end‐expiration. Cardiac and respiratory gating were monitored throughout. Standard cardiac plane images were acquired using the following protocol: (1)Steady‐state free precession (SSFP) cine imaging: Acquired in two‐chamber, short‐axis two‐chamber, three‐chamber, and four‐chamber long‐axis views. Parameters: TE 1.28 ms, TR 2.6 ms, inversion time 200 ms, flip angle 45°, FOV 350 × 305 mm, reconstruction matrix 256 × 192, slice thickness 8 mm. (2) Segmented phase‐sensitive inversion‐recovery (PSIR) sequence: LGE images acquired in four‐chamber and short‐axis planes during diastole 10–15 min post‐intravenous injection of gadodiamide (Magnevist, Bayer, Germany; 0.2 mmol/kg) via power injector. Inversion time was individually optimized (280–350 ms). Parameters: TE 2.99 ms, TR 6.11 ms, inversion time 0 ms, FOV 300 × 300 mm, slice thickness 10 mm.

### 2.3 Cardiac MRI Post‐Processing and Analysis

Image post‐processing and analysis were performed using CVI42 software (Version 6.0.2, Circle Cardiovascular Imaging Inc., Calgary, Alberta, Canada). Image quality underwent initial visual assessment for clarity and artifact presence. Images were classified as poor quality when insufficient clarity or significant artifacts compromised myocardial visualization and accurate analysis. (1) Cardiac Function Analysis: On the short‐axis cine stack, the software automatically delineated LV endocardial and epicardial borders throughout the cardiac cycle, followed by manual correction. Papillary muscles were systematically excluded. The software generated LV ejection fraction (LVEF), LV end‐diastolic volume (LVEDV), and LV end‐systolic volume (LVESV). LVEDV and LVESV were indexed to body surface area and expressed as LV end‐diastolic volume index (LVEDVi) and LVESVi, respectively. On the four‐chamber cine view, left atrial (LA) endocardial and epicardial borders were manually traced at maximal and minimal volumes. The software calculated LA maximal volume, LA minimal volume, and LA ejection fraction (LAEF). (2) LGE Analysis: Short‐axis LGE images were imported into the analysis module. A reference signal baseline was established using visually normal myocardium within the same slice. LGE was defined as regions exhibiting signal intensity > 5 SD above the mean signal intensity of reference normal myocardium [17]. The software automatically marked hyperenhanced regions meeting this threshold and calculated LGE extent (expressed as percentage of LV mass). Reference normal myocardial regions were confirmed through blinded review by two physicians, each with >5 years of CMR interpretation experience. (3) Myocardial Strain Analysis: The software automatically delineated endocardial and epicardial borders on end‐diastolic short‐axis, two‐chamber, three‐chamber, and four‐chamber cine images, tracking these contours throughout the cardiac cycle. Manual correction was performed when software tracking was inaccurate. The following peak systolic global myocardial strain [18]. All images were independently analyzed by two radiologists (each with > 5 years of cardiac imaging experience) blinded to clinical data. One reader performed repeated measurements at 1‐month intervals to assess intra‐observer variability (Figure 2).

**Figure 2:**
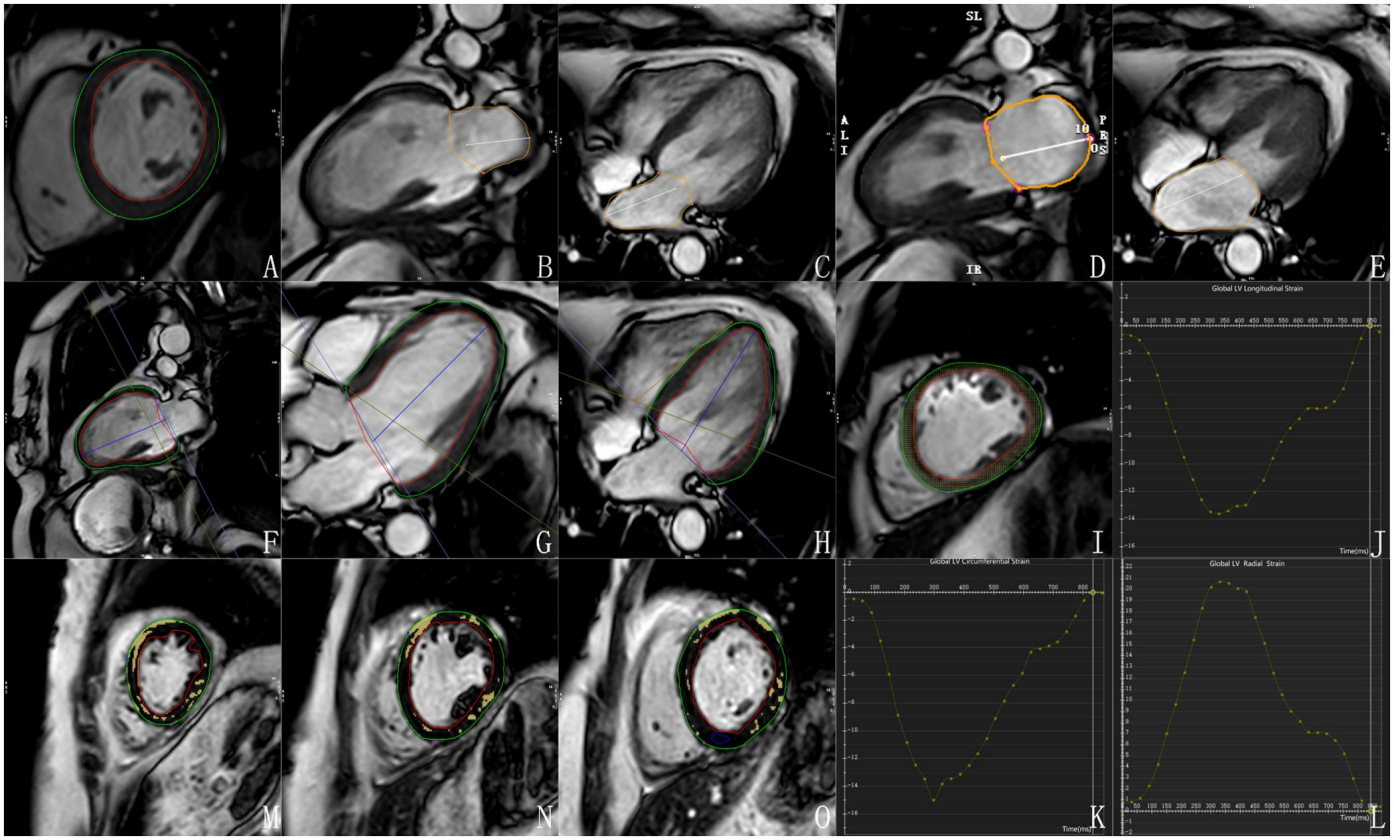
Example MRI studies from a 43‐year‐old female patient hospitalized for heart failure 20 months post‐CMR. (A) LV function calculated from continuous cine short‐axis views. Maximum/minimum LA volumes were calculated using the biplane area‐length method, measuring LA area(orange outlines) and length (white lines) in (B) two‐chamber and (C) four‐chamber views at end‐systole, and in (D) two‐chamber and (E) four‐chamber views at end‐diastole. Global radial strain (GRS), global circumferential strain (GCS), and global longitudinal strain (GLS) were obtained by tracing endocardial(red) and epicardial(green) borders on (F) two‐chamber, (G) three‐chamber, and (H) four‐chamber long‐axis views, and on (I) cine short‐axis images throughout the cardiac cycle. Corresponding strain curves demonstrated GRS 19.07% (J), GCS −15.03% (K), and GLS −8.60% (L). LGE burden was quantified by tracing endocardial(red) and epicardial (green) borders on serial LV short‐axis LGE images (M−O). Regions with signal intensity > 5 SD above normal myocardium were defined as LGE. Yellow shading represents quantified LGE areas; blue circles delineate normal myocardium.

## 3 Statistical Analysis

Data analysis was performed using SPSS software (version 27.0) and R statistical software. Following the general rule of thumb for Cox regression models, a minimum of 10 events per predictor variable was required to ensure adequate sample size [19]. With 46 observed MACE events in this cohort, we could include up to 4 pre‐specified candidate predictors. Quantitative data are presented as mean ± SD for normally or approximately normally distributed data; skewed quantitative data are presented as median (interquartile range [IQR]); categorical data are presented as frequencies (*n*, %). Independent samples *t*‐tests were used to compare normally distributed quantitative data between groups. The Mann−Whitney *U* test was used for non‐normally distributed data or when variances were unequal. Categorical variables were compared using the Chi‐square test or Fisher's exact test, as appropriate. Multicollinearity among independent variables was assessed using the variance inflation factor (VIF), with VIF > 5 indicating significant multicollinearity; collinear variables were excluded. Receiver operating characteristic (ROC) curve analysis determined optimal cut‐off values for quantitative variables using the Youden index (sensitivity + specificity − 1) to maximize discrimination. Inter‐ and intra‐observer agreement was evaluated via intra‐class correlation coefficient (ICC), where ICC > 0.75 indicated good agreement.

Kaplan‐Meier survival estimates were calculated based on optimal cut‐off values, with between‐group differences assessed by log‐rank test. Cox PH regression models evaluated the impact of clinical and imaging parameters on DCM prognosis. The model is defined as:

$$h(\text{t|}X)=h_{0}(t)∙\text{exp}(\beta_{1}x_{1}+\beta_{2}x_{2}+∙∙∙+\beta_{p}x_{p})$$

In the Cox model, *h*(*t* | *X*) = h0(*t*)·exp(βX), where *h*(*t* | *X*) is the hazard function at time *t* given covariates *X*, h0(*t*) is the baseline hazard function, and *β* represents the regression coefficients for each variable. Univariable Cox models first tested associations between MACE and baseline/imaging parameters, reporting unadjusted hazard ratios (HRs) and 95% confidence intervals (95% CIs). This identified several candidate MACE‐associated variables. Variables closely correlated with other candidates (LVESVi with LVEDVi; GCS with GLS) were excluded for multicollinearity, leaving a pre‐specified set of 6 clinically relevant candidates (age, smoking, LVEDVi, GLS, LGE presence, and LGE extent). To address residual collinearity and avoid overfitting, the Least Absolute Shrinkage and Selection Operator (LASSO) regression was applied to these candidate variables. The optimal penalty coefficient λ was selected via 10‐fold cross‐validation using the λ.1se criterion (balancing model simplicity and predictive accuracy). Variables with non‐zero coefficients (LVEDVi, LGE extent) were included in the final multivariable Cox model, reporting adjusted HRs and 95% CIs. Schoenfeld residual tests assessed the proportional hazards (PH) assumption before model fitting. LVEDVi satisfied the PH assumption (*p* = 0.348), while for LGE extent the Schoenfeld test was borderline (*p* = 0.066); given the highly significant LGE extent × time interaction (likelihood ratio test *p* < 0.001), an extended Cox model with a time‐dependent interaction term was retained: $h\left(\text{t|X}\right)h_{0\left(t\right)}$

$$h(\text{t|}X)=h_{0}(t)∙\text{exp}(\beta_{1}\text{LGE\_extent}+(\beta_{2}\text{LGE\_extent}\times \text{time})+\beta_{3}\text{LVEDVi})$$

In this extended model, βLGE represents the effect of LGE extent at baseline (*t* = 0), βLGE × time reflects the dynamic change in the risk effect of LGE extent over time *t*, and βLVEDVi represents the constant hazard effect of LVEDVi. The interaction term's necessity was confirmed by a significant Likelihood Ratio Test (*χ* ^2^ = 13.36, *p* < 0.001).

## 4 Results

### 4.1 Study Population Characteristics

A total of 234 consecutive patients were initially screened. Sixteen patients were excluded due to poor cardiac MRI quality, 30 were lost to follow‐up, and 15 were excluded for ischemic cardiomyopathy. The final cohort comprised 173 patients(age 47 ± 15 years; 61% males). Medical histories included smoking (*n* = 52), alcohol consumption (*n* = 19), hypertension (*n* = 54), and diabetes (*n* = 15). 43% of patients were NYHA class III. Baseline clinical characteristics and conventional cardiac function parameters are detailed in Table 1.

**Table 1:** Baseline characteristics and left ventricular function of patients with DCM.

Variable	ALL patients (*n* = 173)	No MACE Event (*n* = 127)	MACE event occurred (*n* = 46)	Statistic	*p* Value
Age (year)*	47.39 ± 15.02	44.97 ± 14.51	54.09 ± 14.48	3.658	< 0.001
Male	105 (61)	75 (59)	30 (65)	0.538	0.463
Height (cm)*	168.64 ± 8.44	168.63 ± 8.65	168.67 ± 7.92	0.031	0.975
Weight (Kg)*	69.81 ± 13.99	69.31 ± 14.30	71.18 ± 13.13	0.808	0.422
Body mass index (Kg/m^2^)*	24.42 ± 3.77	24.24 ± 3.81	24.94 ± 3.63	1.104	0.273
Body surface area(m^2^)*	1.83 ± 0.21	1.82 ± 0.22	1.85 ± 0.20	0.843	0.402
Heart rate (beats/min)*	81.79 ± 16.27	82.04 ± 16.24	81.09 ± 16.51	−0.337	0.737
Smoking	52 (30)	33 (26)	19 (41)	3.770	0.052
Alcohol consumption	19 (11)	14 (11)	5 (11)	0.001	0.977
Hypertension	53 (31)	37 (29)	16 (35)	0.507	0.476
Type 2 diabetes	15 (9)	12 (9)	3 (7)	0.365	0.546
NYHA class				6.242	0.044
II	55 (32)	47 (37)	8 (17)		
III	75 (43)	52 (41)	23 (50)		
IV	43 (25)	28 (22)	15 (33)		
LVEDVi (mL/m^2^)^†^	128.50 (98.75, 160.48)	122.76 (94.61, 155.13)	141.44 (111.26, 175.68)	1.812	0.070
LVESVi (mL/m^2^)^†^	99.45 (60.84, 133.61)	97.66 (54.18,129.29)	110.56 (76.83, 147.22)	1.769	0.077
LVEF (%)^†^	24.15 (16.38, 38.43)	23.76 (16.31, 39.44)	24.62 (17.87, 30.79)	−0.337	0.738
LVSV (mL)^†^	51.72 (37.59, 73.35)	51.72 (35.55, 78.62)	52.23 (39.83, 68.62)	−0.038	0.971
LVCO (L/min)^†^	3.93 (2.95, 5.29)	3.92 (2.74, 5.38)	3.96 (3.23, 5.04)	0.499	0.618
LAVi‐min (mL/m^2^)^†^	20.06 (13.28, 30.73)	19.91 (13.40, 30.49)	22.73 (13.12, 33.53)	0.474	0.637
LAVi‐max (mL/m^2^)^†^	37.35 (29.29, 49.44)	37.14 (29.52, 47.80)	38.98 (29.33, 56.46)	0.389	0.699
LAEF (%)*	43.44 ± 15.41	43.97 ± 15.12	41.97 ± 16.27	−0.725	0.470

*Note:* Unless otherwise specified, data are numbers of patients with percentages in parentheses. Data are means ± SDs. †Data are medians, with IQRs in parentheses.

Abbreviations: CO, cardiac output; DCM, dilated cardiomyopathy; LAEF, left atrial ejection fraction; LAMVi, minimum left atrial volume index; LAVi, maximum left atrial volume index; LVEDVi, LV end‐diastolic volume index; LVEF, LV ejection fraction; LVESVi, LV end‐systolic volume index; NYHA, New York Heart Association; SV, stroke volume.

### 4.2 MACE Events During Follow‐up

During a mean follow‐up of 27 ± 16 months, 46 patients (26.6%) experienced MACE: cardiac death (*n* = 6, 13.0%), HF rehospitalization (*n* = 29, 63.0%), sustained ventricular tachycardia (*n* = 8, 17.4%), and non‐fatal ventricular fibrillation (*n* = 3, 6.5%).

### 4.3 Comparison of Clinical and MRI Parameters Between MACE and Non‐MACE Groups

No significant differences were observed in LVEDVi, LVEF, LA minimal volume, or LA maximal volume (*p* > 0.05). The MACE group demonstrated significantly more impaired global longitudinal strain (GLS) (median [IQR]: − 5.15% [ −6.61, − 3.83] vs.−5.93% [−9.25, −4.79], *p* = 0.008). Non‐ischemic pattern LGE was present in 59 patients (34.1%), with significantly higher prevalence in MACE patients (32/46 [69.6%] vs. 27/127 [21.3%], *p* < 0.001). LGE extent (%LVmass) was substantially greater in the MACE group (median [IQR]: 7.56% [0, 10.28] vs. 0% [0, 0.0], *p* < 0.001). LV myocardial strain and delayed enhancement parameters are detailed in Table 2.

**Table 2:** Myocardial strain and late gadolinium enhancement characteristics.

Variable	ALL patients (*n* = 173)	No MACE event (*n* = 127)	MACE event occurred (*n* = 46)	Statistic	*p* value
GRS	12.49 (7.71,21.35)	12.74 (7.87,23.02)	11.63 (7.06,20.49)	−1.366	0.172
GCS	−7.72 (−12.03,−5.77)	−7.94 (−12.60,−5.96)	−7.13 (−10.30,−5.14)	1.799	0.072
GLS	−5.72 (−8.60,−4.57)	−5.93 (−9.25,−4.79)	−5.14 (−6.61,−3.83)	2.666	0.008
LGE present*	59 (34)	27 (21)	32 (70)	35.062	< 0.001
LGE percentage of LV mass	0 (0, 3.93)	0 (0, 2.18)	7.74 (0, 11.67)	−5.959	< 0.001

*Note:* Unless otherwise specified, data are medians, with IQRs in parentheses. *Data are numbers of patients with percentages in parentheses.

Abbreviations: GCS, global circumferential strain; GLS, global longitudinal strain; GRS, global radial strain; LGE, late gadolinium enhancement.

### 4.4 Predictive Value of MRI Parameters for MACE and Time‐Varying Attenuation of Risk Effect

Both LVEDVi and LGE extent (including its baseline effect and time interaction effect) demonstrated significant associations with MACE events (*p* < 0.05). Crucially, the risk effect of LGE extent exhibited significant time‐dependent attenuation, indicating an initially elevated risk that attenuated over time. ROC curve analysis identified 6.4% as the optimal LGE extent cut‐off value (AUC = 0.809; sensitivity 0.652, specificity 1.000; Figure 4). Kaplan−Meier survival curves confirmed significantly higher MACE rates in patients with LGE extent ≥ 6.4% vs. <6.4% (log‐rank test: *p* < 0.001, Figure 5).

In univariable Cox regression analysis, age, smoking history, LVEDVi, GLS, LGE positivity, and LGE extent were all significantly associated with MACE (all *p* < 0.05). Multivariable Cox regression incorporating the time interaction revealed that LVEDVi, LGE extent, and the LGE extent×time interaction term were independently and significantly associated with MACE (all *p* < 0.05). For every 10 mL/m^2^ increase in LVEDVi, the risk of MACE increased by 8% (HR = 1.008, 95% CI: 1.003–1.014, *p* = 0.001). The baseline effect (at time zero) showed that for every 1% increase in LGE extent, the risk of MACE increased by 24% (HR = 1.243, 95% CI:1.162–1.329, *p* < 0.001). The significant LGE extent × time interaction term (HR = 0.993, 95% CI: 0.989–0.997; *p* = 0.001) indicated progressive weakening of LGE extent's risk effect over time (Table 3).

**Table 3:** Univariate analysis and time‐dependent Cox proportional hazards regression model (including time interaction terms).

Variable	Univariable Analyses			Multivariable Analyses		
	Hazard ratio	95% CI	*p* value	Hazard ratio	95% CI	*p* value
Age	1.036	1.016–1.058	< 0.001			
Male	1.211	0.659–2.223	0.538			
BMI	1.038	0.968–1.112	0.296			
Smoking	1.827	1.014–3.291	0.045			
Alcohol consumption	1.114	0.439–2.826	0.821			
Hypertension	1.143	0.622–2.097	0.667			
Type2 diabetes	0.665	0.206–2.144	0.494			
LVEF	0.983	0.962–1.004	0.112			
LVEDVi (per 10 mL/m^2^)	1.007	1.002–1.012	0.005	1.088	1.035–1.144	0.001
LAVi‐min	1.007	0.989–1.024	0.448			
LAVi‐max	1.007	0.993–1.021	0.352			
LAEF	0.993	0.974–1.012	0.470			
GRS	0.970	0.939–1.001	0.060			
GCS	1.098	1.022–1.179	0.010			
GLS	1.197	1.064–1.347	0.003			
LGE present	5.139	2.740–9.637	< 0.001			
LGE percentage of LV mass	1.102	1.075–1.130	< 0.001	1.243	1.162–1.329	< 0.001
LGE percentage of LVmass*Time	—	—	—	0.993	0.989–0.997	0.001

*Note:* There were 173 patients, with 46 events in total.

Abbreviation: 95%CI, 95% Confidence Interval; BMI, body mass index; DCM, dilated cardiomyopathy; GCS, global circumferential strain; GLS, global longitudinal strain; GRS, global radial strain; LAEF, left atrial ejection fraction; LAVi‐max, Left atrial maximum volume index; LGE, late gadolinium enhancement; LVCO, LV cardiac output; LVEDVi, LV end‐diastolic volume index; LVEF, LV ejection fraction; LVESVi, LV end‐systolic volume index; LVSV, LV stroke volume; NYHA, New York Heart Association.

The dynamic risk curve for LGE extent illustrates this attenuation: the HR was 1.243 at baseline. By 12 months of follow‐up, the HR decreased to 1.148; at 24 months it was 1.061, and by 30 months it approached unity (HR = 1.020), crossing the null at approximately 34 months (Figure 3). Inclusion of the time interaction term significantly improved model fit (Likelihood Ratio Testχ^2^=13.36, *p* < 0.001, Table 4).

**Figure 3:**
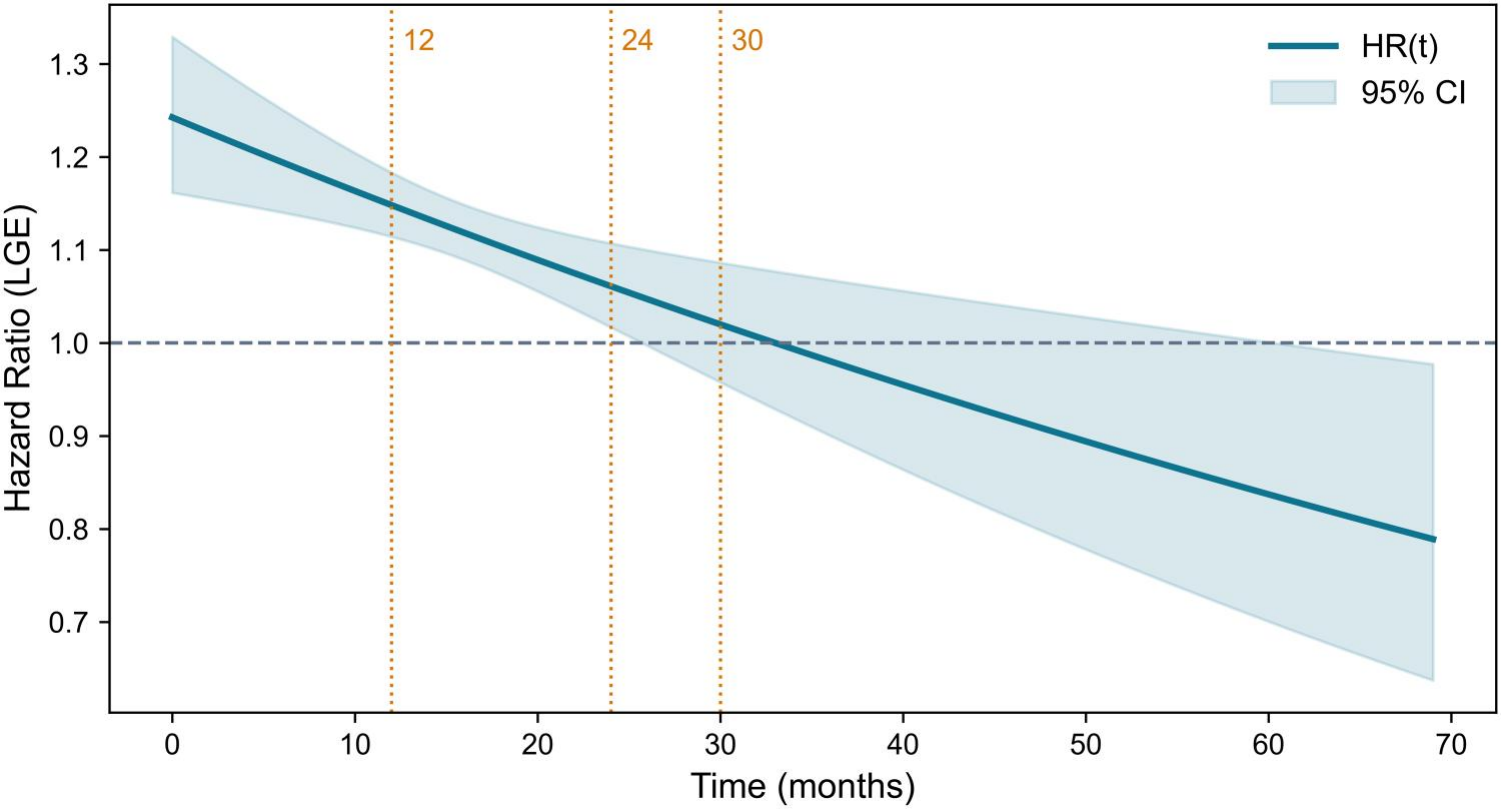
Dynamic curve of hazard ratio for LGE Extent. Shaded area = 95% CI; horizontal dashed line = HR1.0. Vertical dotted lines indicate 12, 24, and 30 months.

**Figure 4:**
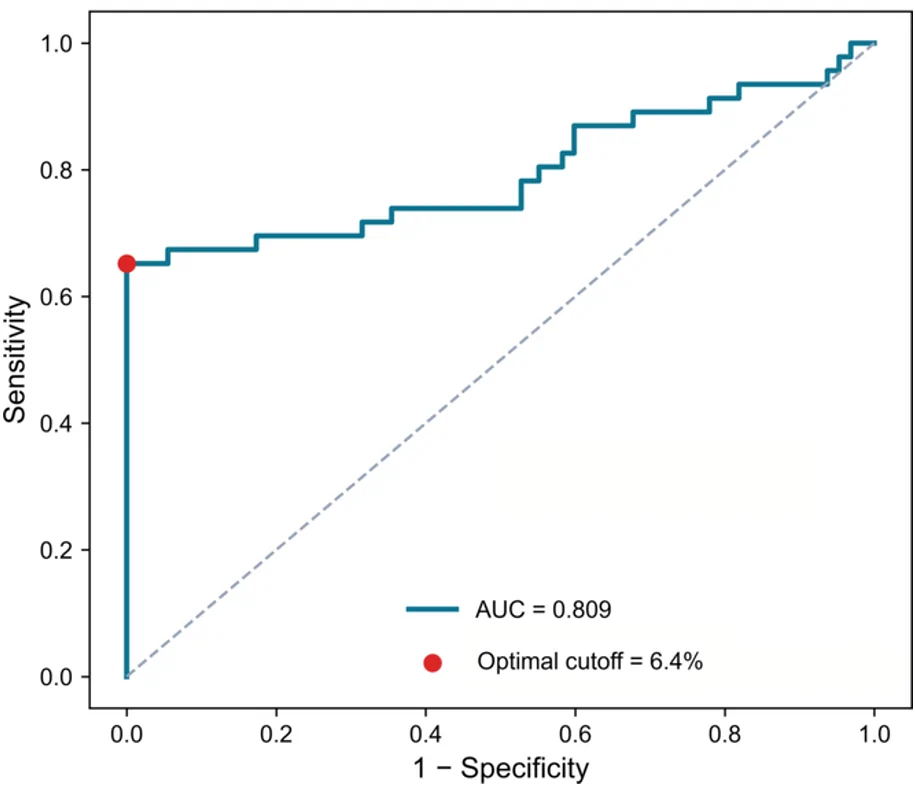
Receiver operating characteristic (ROC) curve for LGE extent predicting MACE. AUC = 0.809; red dot indicates the optimal cut‐off of 6.4%(sensitivity 0.652, specificity 1.000).

**Figure 5:**
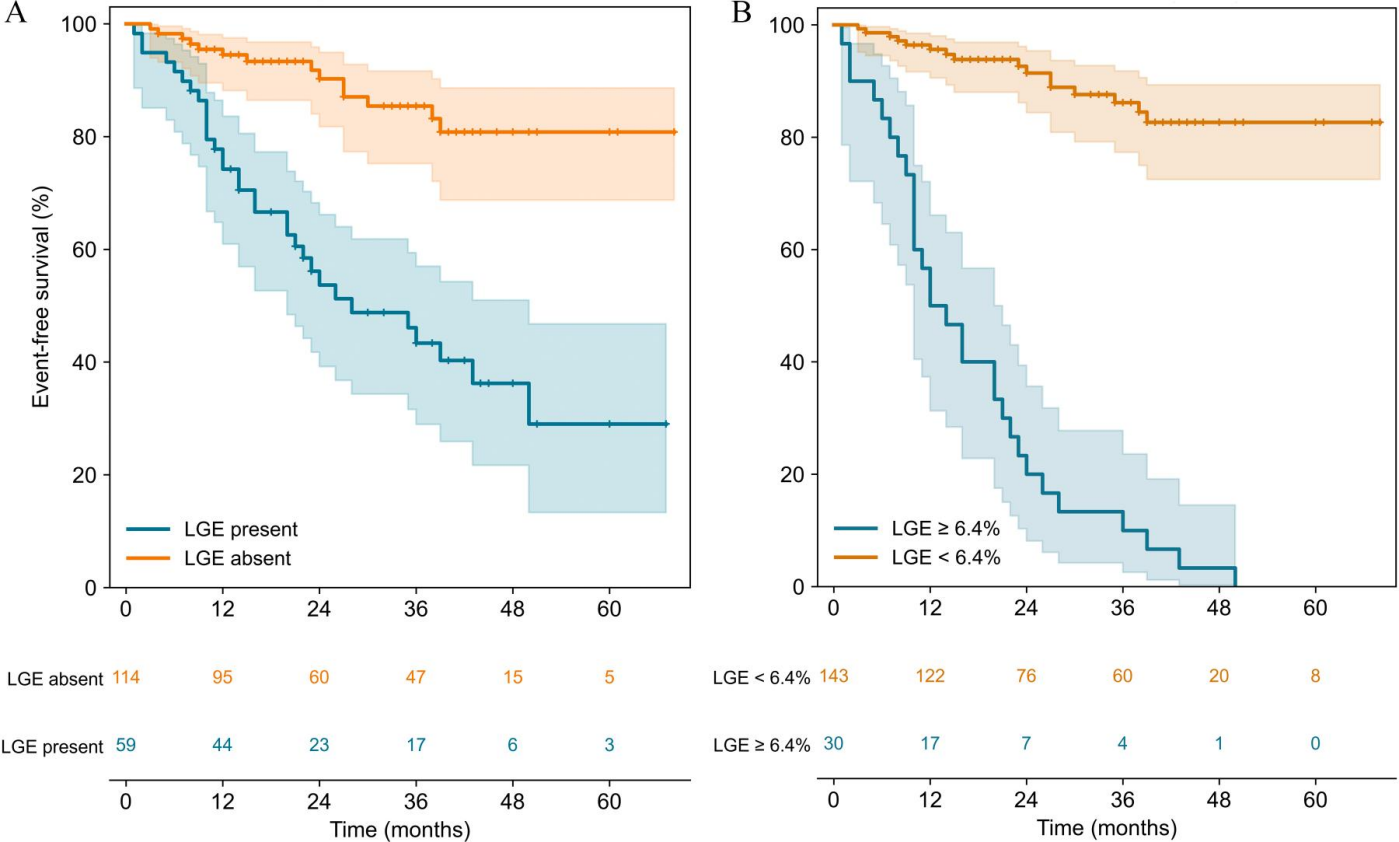
Kaplan‐Meier curve of mace events according to (A) late gadolinium enhancement present (LGE), (B) LGE percentage of LV mass.

**Table 4:** Likelihood ratio test: comparison between unadjusted and extended models.

Model	Chi‐square value	*p* value
Unadjusted model versus extended model	13.360	< 0.001

*Note:* Unadjusted Model: Cox proportional hazards regression for the LGE extent was performed without including time interaction terms, analyzing only the basic association between LGE extent and outcomes; Extended Model: Built on the unadjusted model, with the addition of the “LGE Extent*Time” interaction term to test the time‐dependent effects of LGE.

### 4.5 Inter‐ and Intra‐Observer Agreement Analysis

Inter‐observer and intra‐observer agreement for quantitative measurements of LV functional parameters, myocardial strain indices, and LGE extent were evaluated in a randomly selected subset of 30 study participants. The analysis demonstrated good‐to‐excellent reproducibility for all assessed parameters, with detailed agreement statistics presented in Table 5.

**Table 5:** Inter‐ and intra‐ observer consistency analysis of CMR parameters.

Parameter	Inter‐observer		Intra‐observer	
	ICC	95%CI	ICC	95%CI
LVEDVi	0.950	0.899–0.976	0.996	0.991–0.998
LVESVi	0.917	0.828–0.961	0.996	0.974–0.999
LVEF	0.980	0.958–0.990	0.958	0.911–0.980
LAVi‐min	0.992	0.983–0.996	0.994	0.987–0.997
LAVi‐max	0.996	0.992–0.998	0.997	0.994–0.999
LAEF	0.985	0.969–0.993	0.982	0.963–0.991
GRS	0.964	0.926–0.982	0.955	0.907–0.978
GCS	0.870	0.673–0.944	0.871	0.706–0.941
GLS	0.915	0.800–0.962	0.902	0.806–0.952
LGE percentage of LV mass	0.990	0.979–0.995	0.992	0.984–0.996

Abbreviations: 95%CI, 95% confidence interval; ICC, intraclass correlation coefficient; GCS, global circumferential strain; GLS, global longitudinal strain; GRS, global radial strain; LAEF, left atrial ejection fraction; LAVi‐max, left atrial maximum volume index; LAVi‐min, left atrial minimum volume index; LGE, late gadolinium enhancement; LVEDVi, LV end‐diastolic volume index; LVEF, LV ejection fraction; LVESVi, LV end‐systolic volume index.

## 5 Discussion

This study employed a time‐dependent Cox regression model to investigate independent predictors of MACE, encompassing cardiac death and HF rehospitalization, in patients with DCM. A key finding was the potential time‐dependent attenuation of the risk effect associated with LGE extent. The primary results are as follows: First, the time‐dependent Cox model demonstrated a significant association between LGE extent and MACE. The baseline risk effect was significant (indicating a 24% increased early risk per 1% increase in LGE extent), and the risk effect exhibited time‐dependent attenuation (time interaction term *p* < 0.001). This dynamic characteristic provides new evidence for temporal heterogeneity in the pathological progression of fibrosis. Second, the LVEDV index (LVEDVi), as a quantitative indicator of ventricular remodeling, was independently and positively associated with MACE risk.

Previous studies have established that LGE characteristics (presence and extent) are independent predictors of all‐cause mortality in DCM patients [20, 21, 22, 23, 24]. A meta‐analysis incorporating seven studies indicated that patients with mid‐wall LV fibrosis had a significantly higher risk of SCD or aborted SCD events [25]. Another meta‐analysis showed that DCM patients with LGE had a higher risk of all‐cause death, HF, or SCD events compared to those without LGE [26]. In a recent CMR cohort study of non‐ischemic cardiomyopathy, Pastorini et al. reported that native T1 mapping and extracellular volume (ECV) identified a high‐risk subgroup for arrhythmic events beyond LVEF and LGE [27], supporting the complementary role of quantitative tissue characterization. Consistent with prior conclusions, this study confirmed the significant association between LGE characteristics and MACE, with patients exhibiting an LGE extent ≥ 6.4% experiencing higher MACE rates. More importantly, the time‐dependent Cox regression analysis revealed that the risk effect of LGE extent on MACE attenuated over time. In the early stages of myocardial fibrosis formation, the disordered deposition of collagen fibers causes significant distortion of the myocardial structure and disrupts gap junctions between cardiomyocytes, creating anisotropic conduction pathways that substantially increase the risk of reentrant arrhythmias [28]. Concurrently, the activated renin‐angiotensin‐aldosterone system (RAAS) within fibrotic regions can amplify this risk by promoting cardiomyocyte apoptosis and interstitial fibrosis, forming a vicious cycle [29, 30]. Our study found that the HR associated with LGE extent attenuated from 1.243 at baseline to 1.061 at 24 months and crossed unity at approximately 34 months. This phenomenon may be attributed to the reduced density of voltage‐gated ion channels within mature, stable scar tissue formed later, diminishing its disruptive effect on myocardial electrophysiology [31, 32]. Furthermore, long‐term intervention with beta‐blockers and angiotensin‐converting enzyme inhibitors (ACEIs) can reduce sympathetic tone, potentially weakening the pro‐inflammatory microenvironment of fibrosis and thereby further attenuating its risk effect [33, 34].

LV dilation, the hallmark manifestation of LV remodeling, represents a compensatory mechanism by the heart to maintain LVSV and LVCO. Its progressive trajectory serves not only as a critical indicator of HF progression but also as a primary therapeutic target [35, 36]. Previous research has demonstrated a strong association between progressive worsening of LV dilation and significantly increased risks of all‐cause mortality and the need for cardiac transplantation [37]. Pathophysiologically, increased ventricular stiffening due to myocardial fibrosis shifts the end‐diastolic pressure‐volume curve leftward, compromising diastolic filling mechanics and necessitating increased end‐diastolic volume to preserve pumping function [38], creating a self‐perpetuating cycle of “fibrosis—increased stiffness—volume expansion.” Additionally, abnormal electrical conduction in fibrotic regions precipitates arrhythmias; recurrent electrical disturbances increase ventricular wall stress, stimulating ventricular dilation. Simultaneously, progressive increases in LVEDVi induce mechanical stretch on borderzone myocardium adjacent to fibrotic scars, exacerbating local conduction heterogeneity [39]. GLS is the most widely used myocardial strain parameter in clinical practice. One meta‐analysis indicated no significant association between GLS (per 1% reduction) and cardiac death or HF events (HR = 1.06, 95% CI: 0.95–1.18, *p* = 0.15) [40]. However, other studies suggest that in DCM patients with LVEF > 35%, reduced GLS is associated with increased risks of all‐cause death and HF hospitalization15. Our results showed that while no definitive association was found between GLS and MACE, baseline GLS was significantly lower in the MACE group compared to the non‐MACE group. This discrepancy might relate to the relatively severe impairment of systolic function in this DCM cohort; GLS may hold greater value for risk stratification in the early compensated stages of HF, while its sensitivity decreases in the late stages of severe remodeling, leading to a risk signal dilution.

This study has several limitations. First, as a single‐center retrospective study lacking an independent external validation cohort, the generalizability of the findings may be constrained. Second, given the borderline deviation of the PH assumption for LGE extent (Schoenfeld *p* = 0.066) and the highly significant LGE × time interaction, a time‐dependent Cox regression model was employed instead of the traditional Cox model. Although this method accurately captures time‐dependent risk attenuation, the inclusion of a time interaction term increases the complexity of parameter estimation, requiring larger sample sizes to verify model stability. Third, only baseline LGE extent was included, without tracking the dynamic evolution of LGE burden during follow‐up. Fourth, native T1 mapping and ECV quantification were not performed in this cohort; these tissue‐characterization techniques have emerged as promising prognostic markers in DCM and should be incorporated in future studies. Furthermore, variations in the types, doses, and adherence to prescribed medications among patients may introduce confounding effects on the observed pattern of LGE risk attenuation. Future research plans include conducting prospective multicenter cohort studies incorporating serial cardiovascular magnetic resonance (CMR) monitoring to quantify the spatiotemporal evolution of LGE burden, detailed recording of medication regimens and adherence, and assessment of their potential modifying effects on the pattern of LGE risk attenuation. Ultimately, the goal is to construct integrated predictive models incorporating multi‐dimensional indicators based on dynamically updated time‐series data.

In summary, both LVEDVi and LGE extent are independent predictors of MACEs in DCM. LGE extent holds significant prognostic value for early risk (within the first 2 years of follow‐up), with its risk effect demonstrating time‐dependent attenuation. Conversely, LVEDVi maintains stable predictive power throughout long‐term follow‐up, reflecting the persistent impact of ventricular remodeling. Therefore, intensifying early follow‐up and risk‐stratified monitoring in DCM patients, particularly during the early period when LGE‐related risk is highest, represents a reasonable strategy to improve clinical outcomes within critical time windows during disease progression.

## Author Contributions

**Liyuan Ma:** conceptualization, methodology, data curation, formal analysis, writing – original draft. **Yuan Li:** data curation, investigation, writing – review and editing. **Huairong Zhang:** methodology, writing – review and editing. **Yan Zheng:** investigation, data curation. **Pei Wang:** investigation, writing – review and editing. **Yuanyuan Niu:** investigation, writing – review and editing. **Li Zhu:** conceptualization, supervision, funding acquisition, writing – review and editing.

## Conflicts of Interest

The authors declare no conflicts of interest.

## Data Availability

The data that support the findings of this study are available from the corresponding author upon reasonable request.
